# A historical perspective on the role of sensory nerves in neurogenic inflammation

**DOI:** 10.1007/s00281-018-0673-1

**Published:** 2018-04-03

**Authors:** João Sousa-Valente, Susan D. Brain

**Affiliations:** 1 Vascular Biology and Inflammation Section, Cardiovascular School of Medicine and Science, British Heart Foundation Centre of Excellence, King’s College London, Franklin-Wilkins Building, London, SE1 9NH UK; 2 School of Cardiovascular Medicine & Science, Faculty of Life Sciences and Medicine,, King’s College London, Franklin-Wilkins Building,, London, SE1 9NH UK

**Keywords:** Sensory nerves, Neurogenic inflammation, Transient receptor potential channels, Antidromic vasodilation, Neuropeptides

## Abstract

The term ‘neurogenic inflammation’ is commonly used, especially with respect to the role of sensory nerves within inflammatory disease. However, despite over a century of research, we remain unclear about the role of these nerves in the vascular biology of inflammation, as compared with their interacting role in pain processing and of their potential for therapeutic manipulation. This chapter attempts to discuss the progress in understanding, from the initial discovery of sensory nerves until the present day. This covers pioneering findings that these nerves exist, are involved in vascular events and act as important sensors of environmental changes, including injury and infection. This is followed by discovery of the contents they release such as the established vasoactive neuropeptides substance P and CGRP as well as anti-inflammatory peptides such as the opioids and somatostatin. The more recent emergence of the importance of the transient receptor potential (TRP) channels has revealed some of the mechanisms by which these nerves sense environmental stimuli. This knowledge enables a platform from which to learn of the potential role of neurogenic inflammation in disease and in turn of novel therapeutic targets.

## Introduction

### Early history

The term ‘neurogenic inflammation’ is commonly used to describe the concept that sensory nerves participate in inflammation. The neuropeptide-containing sensory nerves involving the cutaneous system are historically defined as primarily the thin slow conducting C-fibres (1.5–0.3 μm) which are unmyelinated and the small (6-11 μm) Aδ-fibres which possess a thin layer of myelin, predominantly forming part of the somatic nervous system [1]. The history of the development of our knowledge of neurogenic inflammation and of the sensory nerves is fascinating, but complex. The discovery pathway is not linear, with several scientists making similar discoveries around the world at the same time. Whilst some have become historically famous, others even though they made a seminal discovery are not. Goltz realised that the sciatic nerve of the dog contained vasodilator fibres, in addition to the normal constrictor nerves [2]. This was also observed by Stricker (1876) who provided evidence of how sensory nerves may contribute to inflammation [3]. He observed that dorsal roots of the dog when stimulated in an antidromic manner caused an increased blood flow in the skin of the area that was innervated by the sensory nerves, through the use of a mercury thermometer placed between the toes [3].

Meanwhile, Sherrington produced some key studies to show the connecting sensory cutaneous fields and their innervation by spinal afferents. He coined the phrase ‘sensory spinal skin-field’, to differentiate from motor spinal reflexes [4]. This finding was supported by Bayliss in 1901 and Langley (1923), with also a realisation by this time that these findings could be expanded to other species such as the cat and frog [5, 6]. Bayliss stated that the findings he described were of ‘rather a revolutionary nature’. It was these key studies and their discussions that led to the definitions of ‘antidromic vasodilation’, as the dorsal roots only have afferent fibres and removal of dorsal roots abolished the vasodilation (also known as neurogenic vasodilation). Thus, sensory nerves mediate vasodilation.

There were some complementary studies ongoing and several key people were working in this area at the time. The studies that revolved around herpes zoster and the resulting cutaneous pathology were brought into context recently by Oaklander [7]. Knowledge was lacking on the distribution in the skin of the afferent nerve fibres that travel from the skin to the dorsal roots of the spinal cord. Whilst many have since worked to link the sensory nerve system to inflammation in addition to pain, since this time, there was a vital observation before this between herpes zoster and the sensory nervous system. It is now established that varicella-zoster virus, a human herpes virus that causes chickenpox, can become latent in trigeminal and dorsal root ganglia, to be reactivated to mediate shingles (herpes zoster) [8]. A neurologist (Sir Henry Head) and pathologist (Campbell) joined together to write a large monograph where they identified herpes zoster as a disease of the nervous system, involving increased blood flow at the lesional site [9]. Sir Henry Head worked in humans with herpes zoster. He provided the foundation knowledge for dermatomes in his studies for his PhD (1893) and later through a monograph that is still widely referred to today [9]. They were aware of criticisms of their work from others, but concluded the location was in the sensory nerves and their dorsal roots and some were more likely to be affected than others. They also tried to understand the accompanying inflammation and degeneration and through this provided a remarkable recorded insight. The Head and Campbell manuscript is still considered classic within the virology field [7, 10]. This brought centre stage the concept that the sensory nerves play a pivotal role in inflammation, although at the time it does not appear to be acknowledged to any great extent by the classical physiologists. The importance of this work is that, not only was it detailed and thorough, but also it soon became impossible to do that type of work, as the science was allowed to go ahead, involving humans in a way that is impossible today. Critical to their detailed analysis, they were able to study people who had died at institutions (including Guy’s Hospital here at King’s), where they were recorded as previously suffering from herpes zoster. They compared locations of lesions and rash from the clinical notes with that of the nerves and ganglion that were found to innervate the patient at post mortem inspection.

These findings were confirmed and extended such that specific areas of the skin were associated with a certain spinal root and was defined as a dermatome, with little overlap, as determined by either anatomical or physiological methods. A remarkable review by Foerster in 1933 discusses how others (e.g. Herringham and Bolk) traced single roots by dissection to the skin [11]. The work was greatly enhanced by that of Sherrington who teased out nerves and defined the dermatomes in monkeys [4]. Their knowledge utilised the vasodilator properties of sensory nerves as defined by Stricker and Bayliss from animal studies [3, 6]. Foerster concluded from his very complete and very visual studies, from 30 years of work as a neurologist, that areas of vasodilation produced by electrical stimulation of posterior roots are similar, but not identical, to anatomical localisation, and relate to the eruption of herpes in that area. Moreover, in terms of nociception, and taking the work of physiologists who were also able to work on animal species, the dermatome area is remarkedly represented by each filament in the root, so if only a few are lost, this has no effect. Post-herpatic pain was difficult, by comparison, to define. Specifically whether the pain was generalised or generated via the central or peripheral nervous system. At a time when the incidence of shingles advances (with increased longevity) and the pain is intense, this remains very topical today [12]. The inflammation is usually relatively acute, but the mechanisms behind the post-hepatic pain that affects many sufferers are debilitating [13].

### Relevance of capsaicin and related chemicals

The next important discovery was that the topical application of mustard oil, an irritant chemical, to the skin increases blood flow and inflammatory swelling. In a historical review by Gabor Jancsó et al. (2009), they state that the less well known Spiess realised in 1906 that mustard oil, extracted from mustard seeds, induced inflammation that he considered to be due to spinal reflexes [14, 15]. However, the work of Bruce is better known and he realised that inflammation and vasodilation in the eye in response to topical mustard oil were not observed when the eye was anaesthetised or the sensory nerves had degenerated and suggested the involvement of axon reflexes and, by definition, an intact sensory nerve supply [16]. This was substantiated in 1918 by Breslauer who realised that mustard oil required intact sensory nerves in the skin [17]. Sir Thomas Lewis played an important part in allowing the mechanisms of neurogenic inflammation to be further understood through his studies into responses to skin injury. He is perhaps best known for his description of this injury as a ‘triple response’. The three components of this response are a wheal, a flare and a local reddening response. This triple response is similar to that observed when histamine is injected into the skin. The wheal response is caused by oedema formation as a result from increased microvascular permeability. The local reddening and the flare are both consequences of increased blood flow. The flare is of particular interest as it arises from an initial stimulus, leading to an area of erythema that spreads further from the site of injection shown to be dependent on an intact nerve supply. The phrase ‘axon reflex’ was coined where an initial stimulus is able to activate nerves leading to a spread of activation, generally considered to be due to nerve collaterals and, in this case, leading to skin vasodilation over quite a large area. The flare was not observed when the sensory nerves had degenerated, providing further proof for the axon reflex theory [18]. An important component of the work by Bayliss and Lewis was their suggestion, not really realised until the later development of elegant histological techniques, that these sensory nerves were localised perivascularly. These nerve endings are ideally situated for the release of the potent vasodilators that they are now known to contain [19].

However, despite the early relevance and interest in neurogenic inflammation, studies on this subject were stunted. Only in the 1960s was there a re-emergence building on earlier findings using mustard oil. Jancso and coworkers [20, 21] undertook mechanistic studies to elucidate the mediators which may be involved in this response, and soon realised that neurogenic inflammation was not inhibited by classic autonomic antagonists. With this, Jancso also highlighted the importance of another related compound, capsaicin, an irritant found in chilli pepper extract, in studying sensory nerves. Capsaicin was shown to have dual roles, where (1) at low concentration, it dose-dependently causes neurogenic inflammation, yet (2) at repeated, higher concentration leads to sensory nerve desensitisation and loss of neurogenic inflammation response [20, 21]. Of note, historically, it would appear that Hӧgyes reported burning to the skin with an extract of paprika (that would contain capsaicin) as early as 1878 and apparently concluded that it acted via sensory nerves [1]. Soon, Jancso defined a range of chemicals which had the ability to activate sensory nerves. Perhaps, most importantly, Jancso was the first to show the selectivity of capsaicin for sensory nerves and the pivotal finding that capsaicin desensitisation was able to reduce pain sensitivity, but not mechanical sensitivity. His work contributed to an important early basic understanding of the links between sensory nerves and the skin. Still today, sensory denervation mediated by capsaicin or related compounds mediated is one of the most common methods to induce sensory depletion or desensitisation. In addition to the close links between sensory nerves and the skin, this depletion technique seemed to be specific for targeting small-fibre sensory nerves throughout the body [22]. Of note, the application of 8% capsaicin patches has been suggested to help the pain control of some patients, with minimal side effects [12, 23]. These findings were key in realising that transient receptor potential (TRP) channels are localised to sensory nerves.

### Discovery of the sensory neuropeptide substance P

**Fig. 1:**
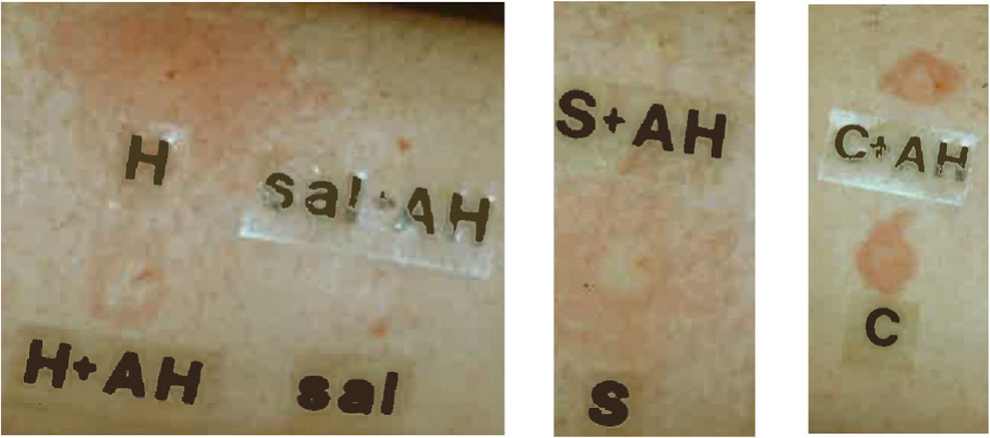
A representative figure to show the effect of co-administration of the histamine H1 receptor antagonist chlorpheniramine (AH, 1 μg/50 μl). The left hand panel shows the inhibitory effect of the antagonist on histamine (H, 500 pmol/50 μl) and saline (sal, 50 μl) injections, the middle panel on substance P (S, 10 pmol/50 μl) and the right hand panel on CGRP (C, 10 pmol/50 μl). Note the positive inhibitory effect on histamine and substance P flare responses, but not on the local reddening induced by CGRP (see also [34])

The discovery of the major neuropeptide substance P took place over several decades. First, Dale suggested that sensory nerves released a transmitter and in studying it realised with Gaddum that it was novel, as not inhibited by any of the then studied major studied mediators (e.g. acetylcholine and histamine). Dale in 1935 considered the sensory transmitters and concluded that transmitters would be released to cause the vasodilation and should be released both antidromically and orthodromically [24]. The ‘P’ referred to a ‘powder’ that they prepared as a purified standard preparation. Gaddum and Schild are considered to have started using the name substance P and von Euler was involved in the extraction from the gut and brain [25]. The peptide structure of substance P was published in 1971 by Susan Leeman [26]. It was realised that capsaicin treatment of the spinal cord depleted substance P [27, 28]. Soon, it was realised that substance P was found throughout the central and peripheral nervous systems, from the work of Hokfelt [29]. Lembeck and colleagues confirmed the importance of substance P in mediating neurogenic vasodilation and increased microvascular permeability [30] updating the Bayliss technique to the rat and counting drips of blood. This response was not observed after capsaicin pretreatment [31, 32] or later when available, in the presence of substance P antagonists [33]. In human skin, substance P has the ability to mediate a histamine-induced flare [34], as shown in Fig. 1.

The word ‘tachykinin’ was developed by Erspamer and the best known members of the family are substance P, neurokinin A and neurokinin B [35]. However, it took some time to gain an understanding of the receptor family through which substance P acts. It is now known that substance P acts in a vasoactive manner predominantly through the neurokinin NK1 receptor and that the NK2 receptor with preference for neurokinin A and the NK3 receptor with preference NK3 also exist [36]. A major problem in unravelling the biology was that the receptors are species selective, such that the first generation of antagonists was not human selective [36]. Eventually, non-peptide NK1 receptor antagonists were developed, with the aim of being new analgesics and anti-inflammatory agents. Whilst these agents were effective in pre-clinical studies involving animal models, they were not in humans. A range of clinical trials including for arthritis and migraine and depression has failed [37]. There is a discussion among tachykinin experts that the NK1 antagonists will benefit pruritus, if only we knew more about the sensory nerves involved. Of note, the clinically used substance P antagonist aprepitant is approved for the treatment of nausea and vomiting induced by chemotherapy and a recent small study has shown that aprepitant benefited refractory pruritus associated with malignancies, inferring the need for further study [38]. Indeed, it is suggested that in total, recent small-scale studies have shown benefit in 110 patients suffering from pruritus given an NK1 antagonist, with larger clinical trials now ongoing [39].

### Discovery of the sensory neuropeptide calcitonin gene-related peptide

The 37 amino acid calcitonin gene-related peptide (CGRP) was discovered using molecular biology techniques, when it was realised that alternative RNA processing of the gene for calcitonin results in the generation of CGRP. CGRP can exist in two forms (α and β), both of which differ minimally in structure and across species. The β form is produced by a separate gene and historically considered to be found in the gut and brain but has been more recently found in vascular sources and they have very similar biological activities [19]. Rosenfeld and colleagues showed that the rat thyroid medulla failed to maintain calcitonin production, when serially transplanted, switching instead to producing GGRP, via the alternative splicing of the gene [3, 6, 40]. Human αCGRP was discovered (34) [33]. It was soon realised when antibodies were produced and immune-histochemical studies performed that whilst CGRP is found in the thyroid of ageing rats and in human carcinoma of the thyroid, it is localised throughout the peripheral and central nervous systems with substance P in sensory nerves [41, 42]. Moreover, CGRP is an extremely potent microvascular vasodilator in the cutaneous circulation [19, 43]. Indeed, it is a vasodilator in most vascular beds, especially also associated with the heart and trigeminal circulations [44]. Evidence that CGRP was involved in cerebral regulation led to the intense study continuing until the present day of its role in migraine [19, 45].

CGRP is now known to be a member of the CGRP family, alongside adrenomedullin and amylin. CGRP acts through the CGRP receptor complex [46, 47]. This complex requires co-localisation of the G-protein component calcitonin-like receptor (CLR) with a single transmembrane component known as receptor activity-modifying protein 1 (RAMP1), and an intracellular signalling component receptor component protein (RCP) [47].

Over the years, some looked by at Thomas Lewis’s work on the axon reflex flare, in an attempt to understand its pharmacological mechanisms. Their research was hindered by the fact that it is not observed to such an extent in typical laboratory species, reviewed by Chapman (1977). Chapman, who studied this phenomenon suggested that a peptide similar to a kinin, neurotensin or substance P is responsible for the vasodilator response [48]. It is now realised that CGRP is most likely the principal mediator. It was shown to mediate the capsaicin-induced flare in humans in studies involving aprepitant, the cyclo-oxygenase inhibitor indomethacin and the nitric oxide synthase inhibitor L-NMMA [49]. CGRP had been known to mediate the cutaneous vasodilation following a low stimulation of the saphenous nerve in the rat; however, substance P mediates the oedema formation in this model upon more intense stimulation in this model [50]. The ability of endogenous substance P to mediate oedema formation in humans is more difficult to ascertain, although exogenous substance P is certainly active as discussed.

The pathological role of CGRP has been sought via development of antagonists and antibodies. This has been driven by evidence that it plays a major role in migraine [45] and possibly other processes associated with pain and itch [19, 51, 52]. CGRP antibodies and antagonists are in late-phase clinical trials for migraine. They have not been extensively investigated in patients with either cardiovascular or skin conditions to date. Indeed, the human administration of CGRP antagonists and antibodies has revealed that this potent vasodilator does not influence cardiovascular regulation in the healthy human. On the other hand, a lack of endogenous CGRP release may enhance cardiovascular disease as shown in CGRP knockout mice [53] and by the finding that a long-acting CGRP agonist benefits cardiovascular dysfunction, including heart failure in the mouse, and CGRP administration is beneficial in human heart failure and other vascular diseases [19, 54, 55]. Thus, this peptide may be a double-edged sword in terms of human physiology and pathology.

### Other neuropeptides

Whilst substance P and CGRP are the best known neuropeptides, a range of others is either synthesised or upregulated to be synthesised in disease. One of these somatostatin was also discovered [56] and later shown to be anti-inflammatory, but despite drug development projects, its role remains unclear [57]. The neuropeptides are primarily synthesised at the dorsal root as larger pro-peptides and then travel antidromically via the neurons to the nerve terminals where they are released as the peptide from vesicles [19]. One of the most interesting aspects is their plasticity in terms of gene regulation in response to injury.

### Sensory nerve activation. TRP receptors

The sensory nerves are activated by a range of environmental and endogenous stimuli, including thermal, mechanical and chemical, and are critically involved in processing nociceptive information [58] whilst providing the neurogenic influence in cardiovascular regulation and inflammation. The acute effects, such as oedema formation and increased blood flow, are involved in the response of the skin or other organs to exposure to environmental irritants. The chronic effects have been more difficult to tease out with many indications of pro- and anti-inflammatory effects of sensory nerves suggested. Overall, we still lack understanding in this area.

#### Transient receptor potential channels

Research initiated by the study of capsaicin led to the realisation that the transient receptor potential vanilloid 1 (TRPV1) is a capsaicin agonist [59]. Moreover, whilst it is found on some non-neuronal tissues, TRPV1 is primarily localised to sensory nerves and mediates noxious heat and thermal hyperalgesia [59–61].

**Fig. 2:**
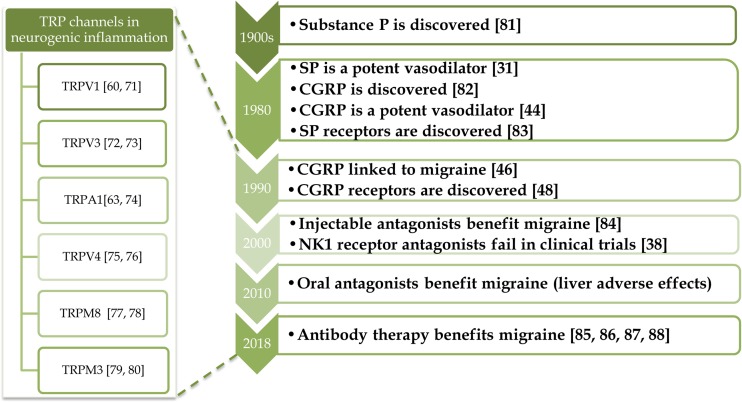
Timeline of neuropeptide research: from bench to bedside and discoveries concerning the involvement of TRP channels in neurogenic inflammation

TRP Ankyrin 1 (TRPA1) channels are found on about 60–75% of TRPV1-positive sensory nerves [62]. These two TRP channels are activated by distinct chemical agents, with TRPV1 mediating noxious heat and TRPA1 mediating cooler temperatures including noxious cold [63, 64], with effects closely linked to temperature-induced vascular changes [65]. There is evidence that both TRPV1 and TRPA1 have important roles in acute and chronic inflammation from knee joint models such as Freund’s complete adjuvant (CFA) [66, 67]. Many pre-clinical studies using laboratory species implicate TRPV1 in hyperalgesia and inflammatory pathways. In comparison, studies using the first generation of TRPV1 antagonists have been less successful as they induce hyperthermia alongside a risk of a lack of awareness of hot surfaces [68]. However, second-generation TRPV1 antagonists are now reported to be in clinical trials that lack this hyperthermia effect [69]. Currently, TRPA1 antagonists are under clinical trials and shown to be beneficial in phase 2 clinical trials for diabetic neuropathy [70]. There are also a myriad of other TRP channels now under investigation (see Fig 2). The TRP family is composed of 28 ligand-gated non-selective cation (e.g. Ca^2+^) ion channels that are composed of subfamilies with defined sensitivities to temperature, chemicals and pressure [58]. This subject is of direct relevance to the contents of this book.

In conclusion, whilst much progress has been made over the years, there is a realisation that we still have many discoveries to make, especially those that relate to the role of these nerves and their activating systems. We find a range of exciting discoveries throughout this book but also many questions that are still unanswered.
